# A bacterial virulence factor interacts with the splicing factor RBM5 and stimulates formation of nuclear RBM5 granules

**DOI:** 10.1038/s41598-022-26037-w

**Published:** 2022-12-19

**Authors:** Renaud Pourpre, Goran Lakisic, Emma Desgranges, Pascale Cossart, Alessandro Pagliuso, Hélène Bierne

**Affiliations:** 1 Université Paris-Saclay, INRAE, Micalis Institute, EpiMic Lab, Jouy-en-Josas, AgroParisTech France; 2 Institut Pasteur, Paris, France

**Keywords:** Cell biology, Microbiology

## Abstract

*L. monocytogenes* causes listeriosis, a foodborne disease that is particularly dangerous for immunocompromised individuals and fetuses. Several virulence factors of this bacterial pathogen belong to a family of leucine-rich repeat (LRR)-containing proteins called internalins. Among these, InlP is known for its role in placental infection. We report here a function of InlP in mammalian cell nucleus organization. We demonstrate that bacteria do not produce InlP under in vitro culture conditions. When ectopically expressed in human cells, InlP translocates into the nucleus and changes the morphology of nuclear speckles, which are membrane-less organelles storing splicing factors. Using yeast two-hybrid screen, immunoprecipitation and pull-down experiments, we identify the tumor suppressor and splicing factor RBM5 as a major nuclear target of InlP. InlP inhibits RBM5-induced cell death and stimulate the formation of RBM5-induced nuclear granules, where the SC35 speckle protein redistributes. Taken together, these results suggest that InlP acts as a nucleomodulin controlling compartmentalization and function of RBM5 in the nucleus and that *L. monocytogenes* has developed a mechanism to target the host cell splicing machinery.

## Introduction

*L. monocytogenes* is a Gram-positive bacterium infecting humans and various animal species, including farm animals. It is the causal agent of a foodborne infection, listeriosis, which can lead to septicemia, meningoencephalitis and spontaneous abortions^1^. This bacterial species is characterized by a cytosolic life phase, during which bacteria replicate and spread from cell to cell by hijacking the actin cytoskeleton of the host, allowing dissemination in tissues^2^. *L. monocytogenes* can also survive within vacuolar compartments, either derived from phagosomes (*i.e.,* SLAPs and eSLAPs^3,4^) or formed de novo in a late phase of the infection process that could promote the persistence of this pathogen in epithelial tissues (i.e., LisCVs^5,6^).

The different stages of the *L. monocytogenes* infection cycle rely on the production of surface-anchored or secreted virulence factors that specifically interact with eukaryotic components located at the plasma membrane, in the cytosol or different organelles^2,7^. *L. monocytogenes* can also target the host cell nucleus by secreting nucleomodulins, a family of proteins that interact with nuclear factors^8^. Three *L. monocytogenes* nucleomodulins have been characterized so far. LntA modulates the interferon response by targeting the epigenetic regulator BAHD1^9,10^, which is a core component of a chromatin-remodeling complex^11,12^. OrfX decreases the antimicrobial response of macrophages by interacting with RYBP, a protein partner of several transcription factors^13^. Zea is a secreted RNA-binding protein that regulates virulence by modulating the RIG-I-dependent interferon response^14^. During *L. monocytogenes* infection, a fraction of Zea translocates to the nucleus, but at present the functional role of the nuclear Zea remains to be addressed.

Among the virulence factors of *L. monocytogenes*, several belong to the multigenic family of internalins, characterized by the presence of a leucine-rich repeat (LRR) domain^15,16^. LRR domains are optimized for protein–protein interactions. They are present in a large number of proteins involved in a wide variety of functions, including adhesion and signal transduction in eukaryotes^17^ and pathogenesis or environmental interactions in prokaryotes^15,18^. In *L. monocytogenes*, the term internalin was chosen after the discovery of Internalin A (InlA) and InlB, two surface-anchored proteins that mediate internalization into non-phagocytic cells^16,19^. Since then, other internalins have been characterized. InlH was shown to affect the production of the cytokine IL-6 during murine listeriosis^20^; InlJ, InlF and InlL play a role in bacterial adhesion^21–23^; InlK counteracts autophagy^24^; InlC has a dual role: on the one hand, in the intercellular dissemination of bacteria via its interaction with the cytoskeleton protein TUBA^25,26^ and, on the other hand, in innate immunity via its interaction with IKKα of the NF-κB pathway^27,28^.

While the majority of internalins are surface proteins, four internalins with a secretory signal peptide (InlC, InlP, Lmo2027 and Lmo2445) lack any known surface association domain and therefore are predicted to be secreted^15^. However, to our knowledge, only InlC has been formally demonstrated to be secreted by bacteria^27,29^. Faralla and collaborators have studied InlP (Lmo2470), which is present only in the pathogenic *L. monocytogenes* species, and its paralog Lmo2027, which is present in both pathogenic and non-pathogenic *L. monocytogenes* species^30,31^. Infection studies in mice using an *inlP*-deficient *L. monocytogenes* mutant revealed a role of InlP in placental infection^30^. In addition, a yeast two-hybrid screen of a human placenta cDNA library highlighted a protein of the intercellular junctions, Afadin, as a binding partner of InlP^31^. When InlP is ectopically expressed in the cytoplasm of epithelial cells, it interacts with Afadin and this interaction facilitates the crossing of *L. monocytogenes* across the basement membrane. It has been proposed that this mechanism stimulates the formation of protrusions on the basal cell surface and promotes bacterial crossing of the maternal–fetal barrier^31^. However, these studies did not show that InlP is produced and secreted by the bacteria.

Here, we establish that InlP is a secreted internalin that is not expressed by bacteria grown in laboratory conditions in vitro. We find that InlP is an additional member of the nucleomodulin protein family as it is able to target the nucleus of mammalian cells. Ectopic expression of InlP strongly affects the structure of the nuclear speckles—the sites where the splicing machinery accumulates^32^—without changing the structure of other nuclear bodies. In addition, InlP interacts with the splicing factor and tumor suppressor RBM5^33–36^ and counteracts the RBM5-induced cell death thus acting as a RBM5 inhibitor. Expression of InlP stimulates the formation of RBM5-induced nuclear condensates, suggesting a role for InlP in RBM5 compartmentalization in the nucleus. Our data expand the repertoire of *L. monocytogenes* nucleomodulins and highlight a novel activity of a bacterial virulence factor.

## Results

### InlP is a secreted internalin

**Figure 1:**
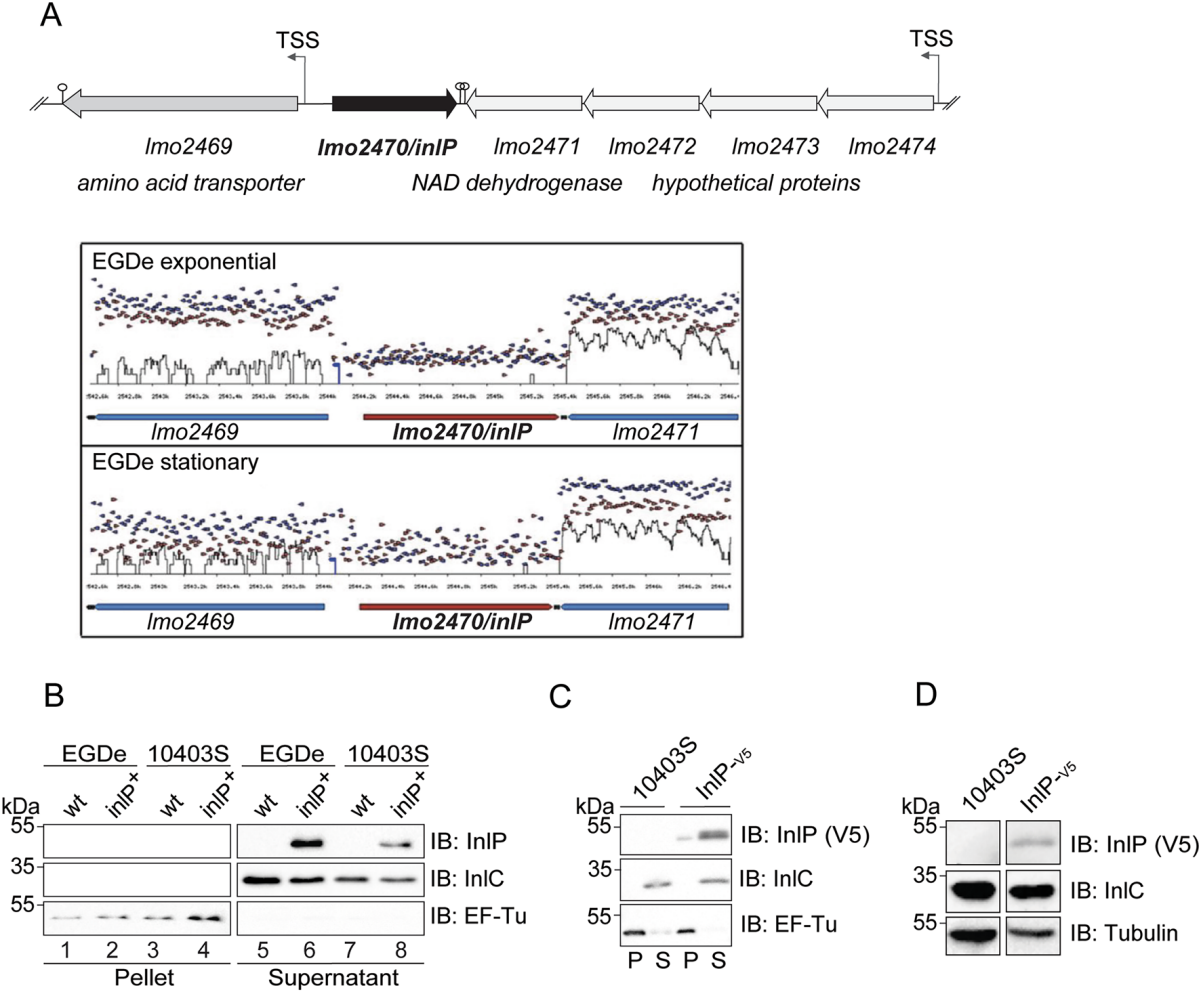
InlP is a secreted protein that is not produced in vitro. (**A**) Genomic organization of the *inlP*-containing genomic locus in *L. monocytogenes* EGDe*.* Arrows and stem and circle represent the transcriptional start sites (TSS) and the transcriptional terminators, respectively. Below, the transcriptional tiling maps of this locus in strain EGDe at exponential or stationary phase in BHI medium at 37 °C (generated from RNA-seq data from^37^). (**B,C**) Analysis of InlP production in bacterial culture pellets and supernatants in wild type EGDe or 10403S strains or in *inlP* constitutively-expressing strains (*inlP* + or InlP-V5). Total bacterial extracts and supernatant precipitates from stationary phase cultures were separated by SDS-PAGE. InlP or InlP-V5 were detected by immunoblotting with an affinity-purified InlP polyclonal antibody (B) or a monoclonal V5 antibody (C), respectively. EF-Tu and InlC antibodies were used to control the pellet (P) and supernatant (S) fractions, respectively. (**B**) Analysis of InlP production in *L. monocytogenes* EGDe- and 10403S-containing plasmid pP1 (wt) or pP1-*inlP (inlP* +*)*. (**C**) Analysis of InlP production in *L.* *monocytogenes* 10403S and 10403S-InlP-V5 strains. (**D**) Analysis of InlP secretion during infection of JEG-3 cells by 10403S-InlP-V5 strain. Cell extracts of JEG-3 cells infected for 8 h were analyzed by immunoblotting with V5 antibody. InlC detection was used as a control for *L. monocytogenes* infection and tubulin, as a control for the amount of cell lysates.

In the *L. monocytogenes* strain EGDe genome, the *inlP* gene (also known as *lmo2470*) is located between *lmo2469* and *lmo2471*, two divergently transcribed genes encoding an amino acid transporter and a NADPH dehydrogenase, respectively (Fig. 1A). Using previous RNA-sequencing (RNA-seq) transcriptomic data from the EGDe strain grown under different conditions^37^, we generated the transcriptional maps of the *inlP* locus, which highlighted that *inlP* had no transcriptional start site under exponential or stationary phase growth conditions in BHI medium at 37 °C. (Fig. 1A). Compared to adjacent genes, *inlP* was not expressed under any of the other growth conditions tested by this study (*i.e.,* at 30 °C, under hypoxic conditions, or in *L. monocytogenes* Δ*sigB* or Δ*prfA* mutant backgrounds)^37^. To determine whether the *inlP* open reading frame encoded a secreted protein, we generated an antibody directed against a peptide located in the C-terminal region of InlP. The antibody detected a recombinant InlP protein fused to the glutathione-S-transferase (InlP-GST) produced in *Escherichia coli* (Supplementary Fig. S1A, S1B). However, consistent with the low *inlP* transcription in *L. monocytogenes* grown in BHI medium (Fig. 1A), the InlP antibody did not detect any protein either in total extracts or culture medium of EGDe or 10403S *L. monocytogenes* strains (Fig. 1B, lanes 1, 3, 5 and 7). We therefore expressed in both strains *inlP* gene under the control of a heterologous promoter (EGDe-InlP + and 10403S-InlP +), which allows constitutive expression in *L. monocytogenes,* as previously described for other virulence factors^9,14,21,24^. Under this condition, InlP was readily detected in the culture medium of both strains grown in exponential or stationary phase (Fig. 1B, lanes 6 and 7; Fig. S1C). For these experiments, bacterial fractions were tested for the presence of the cytoplasmic protein EF-Tu and the secreted protein InlC. It is noteworthy that the precursor of InlP was not detected in the intracellular fraction suggesting that, like InlC, InlP is rapidly secreted after its synthesis (Fig. 1B). In addition, the electrophoretic mobility of InlP was slower than expected, with an apparent molecular weight of about 48 kDa instead of the predicted 39 kDa, which could result from the high content of acidic amino acids (isoelectric point = 3.5), as described for other proteins^38^. Together, these results indicate that when the expression of *inlP* is driven by a constitutive promoter, this gene produces a secreted internalin.

### InlP is not required for *L. monocytogenes* multiplication in human epithelial cells

To investigate the role of InlP during cellular infection by *L. monocytogenes*, we generated two *inlP*-deletion mutant strains (EGDe-Δ*inlP* and 10403S-Δ*inlP*). These InlP-deficient strains were compared to isogenic wild type (wt) strains, as well as constitutively expressing InlP strains, in infection assays of human HepG2 hepatocytes or JEG-3 placental cells. At 2 h, 24 h or 72 h post-infection, there was no significant difference in bacterial entry, multiplication or dissemination between the strains (Fig. S1D). This result prompted us to test whether, under these experimental conditions, InlP secretion or stability was optimal. We thus examined whether secreted InlP could be recovered in the host cells during infection by immunoblotting lysates of infected cells with the InlP antibody. We were unable to detect secreted InlP in infected cells as our antibody recognized several non-specific eukaryotic proteins (not shown). We therefore generated a *L. monocytogenes* strain producing InlP fused to the V5 epitope tag. This strain (*inlP-*_V5_) carries a chromosomally-integrated V5-tagged *inlP* gene under the control of a heterologous promoter (pHyper) in the10403S-Δ*inlP* strain. The V5 antibody detected secreted InlP-_V5_ in the culture medium of the *inlP*-_V5_ strain (Fig. 1C). We then used the *inlP*-_V5_ strain to infect JEG-3 cells and look for the presence of InlP in total cell lysates. We were able to detect InlP in infected cells infected for 8 h, albeit at a very low level (Fig. 1D). In contrast, InlC, a *L. monocytogenes* internalin whose expression is strongly up-regulated in infected cells^27,29^, massively accumulated intracellularly. These results suggest that the secretion and stability of InlP could be tightly regulated, possibly by molecular signals that are not fully recapitulated during infection of cultured cells in vitro.

### InlP ectopically expressed in human cells localizes to the nucleus and alters the shape of splicing speckles

**Figure 2:**
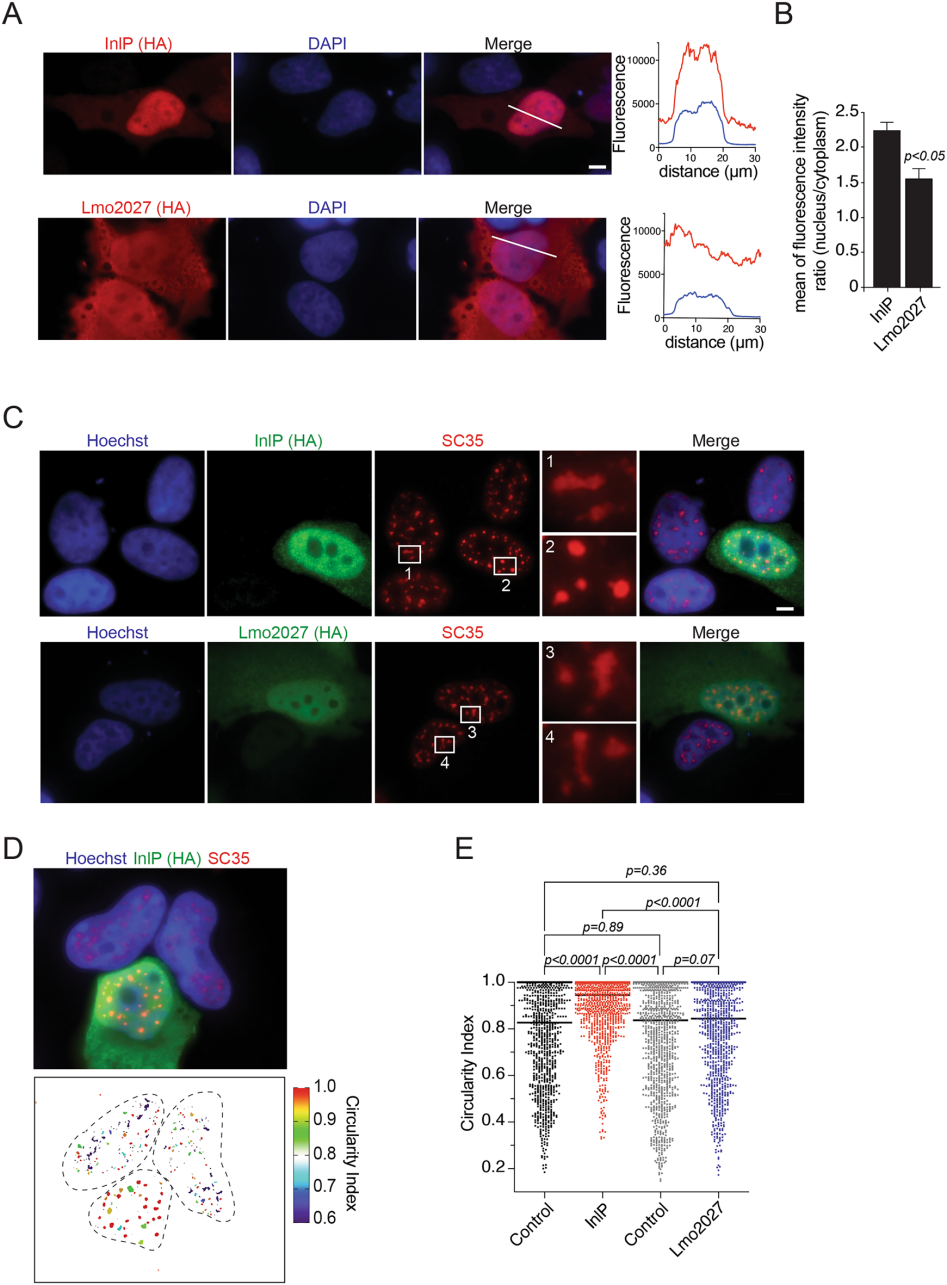
InlP translocates to the nucleus and changes the shape of splicing speckles. (**A**) Left, representative immunofluorescence images of HeLa cells transfected with InlP-_HA_ or Lmo2027-_HA_ and stained with an HA antibody and DAPI.  Right, corresponding pixel intensity plot for white lines drawn on the left. Scale bar 10 µm. (**B**) Nucleus to cytoplasm fluorescence intensity ratios of InlP-_HA_ or Lmo2027-_HA_, as determined with ICY software. Data are mean ± SD of triplicate experiments (n = 22 cells per replicate). (**C**) Immunofluorescence of InlP-_HA_ or Lmo2027-_HA_ and endogenous SC35 in HeLa cells transfected as in (**A**) and stained with HA and SC35 antibodies and Hoechst. Scale bar 10 µm. Representative SC35-positive speckles are enlarged in squares 1 and 4 for non-transfected cells, in square 2 for InlP-transfected cells, and in square 3 for Lmo2027-transfected cells. (**D**) Nuclear speckle circularity in HeLa cells transfected with InlP-_HA_. On the top is a representative image stained as in (**C**) with SC35-positive nuclear speckles in red. Below, the color-coded representation of nuclear speckle circularity index. (**E**) Scatter plot of the circularity index of nuclear speckles from at least 20 cells transfected as in (**C,D**) determined by ImageJ software. The bar represents the median. Statistical significance determined by ANOVA.

In order to understand the function of InlP, while circumventing the problem of its expression, we analyzed its localization in host cells when directly produced by human cells by transfection. We performed the same analysis with Lmo2027, the structural paralog of InlP (65% identity and 77% similarity to InlP^31^), which can be used as a control for InlP-specific effects. HA-tagged InlP or Lmo2027 were expressed in HeLa cells and their localization was revealed by immunofluorescence using an anti-HA antibody, 24 h post-transfection. Strikingly, microscopy analysis highlighted a strong accumulation of InlP in the nucleus. In contrast, Lmo2027 was more evenly distributed in the cell, localizing both in the nucleus and in the cytoplasm (Fig. 2A). Quantification by image analysis of the respective proportion of InlP_-HA_ and Lmo2027_-HA_ in the nucleus versus cytoplasm confirmed that the nuclear InlP signal was significantly higher than that of Lmo2027 (Fig. 2B). Nuclear localization of InlP_-HA_ was also observed in HEK293-FT and LoVo cells ruling out a cell type related phenotype (Fig. S2).

Given the nuclear localization of InlP, we next assessed whether it could affect the spatial distribution of nuclear sub-compartments. The nucleus is organized into distinct chromosomal territories and numerous spheroidal membrane-less organelles (MLO), referred to as nuclear bodies^39,40^. We examined the distribution of SC35, coilin, PML and nucleolin, which are markers of splicing speckles, Cajal bodies, PML bodies, and nucleoli, respectively. While the presence of InlP did not affect PML, coilin or nucleolin labelling (Fig. S3A), it profoundly altered the pattern of the SC35-positive splicing speckles (Fig. 2C). Compared to non-transfected cells, where splicing speckles are structures of variable size and irregular shape, the morphology of splicing speckles massively changed in InlP-expressing cells, forming brighter round-shaped structures (Fig. 2C). Quantification of the circularity of the SC35-positive domains confirmed that InlP expression induced a morphological change in nuclear speckles, which became rounder and less elongated (Fig. 2D,E). Importantly, ectopic expression of Lmo2027 did not affect the morphology of splicing speckles (Fig. 2C,E), demonstrating a specific effect of InlP.

Taken together, these data suggest that InlP acts as as nucleomodulin regulating the morphology and possibly the dynamics of the nuclear speckles.

### InlP interacts with RBM5 and inhibits the pro-apoptotic effect induced by RBM5 overexpression

The nuclear localization of InlP and its effect on nuclear speckle morphology prompted us to assess whether it could interact with nuclear proteins, and in particular splicing factors. This was carried out by a large-scale yeast two-hybrid (Y2H) screen of a human placenta cDNA library, as previously done for other *L. monocytogenes* nucleomodulins^9,13^. To assess the specificity of the interactions, we performed the same screening using Lmo2027 as a bait. Remarkably, among the 14 putative binding partners obtained for InlP, 11 (79%) were nuclear proteins or proteins shuttling between the nucleus and the cytoplasm, in agreement with the localization of InlP (Table 1A). One of the strongest InlP interactors was the RNA-binding motif protein 5 (RBM5), a splicing factor and component of the spliceosome A complex^34,35^. None of the InlP preys were shared with the Lmo2027 putative interactors, indicating a non-overlapping function of the two proteins (Table 1B). It should be noted that Faralla and colleagues also previously identified RBM5 in their Y2H screen for InlP binding partners in placental cells^31^. Importantly, comparison of these two independent screens revealed that only three proteins reproducibly interacted with InlP (Table 1C), including RBM5 with a high interaction score.

**Table 1:** Results of Y2H screens of interactors of InlP or Lmo2027.

Gene symbol	Gene name	Localisation	Score	#clones
**(A) InlP Y2H screen (this study)**				
ZFP1	Zinc finger protein 1	Nucleus	B	7
**RBM5**	**RNA-binding protein 5**	**Nucleus**	**B**	**4**
**DNMT3A**	**DNA (cytosine-5)-methyltransferase 3A**	**Nucleus**	**C**	**4**
THAP5	THAP domain-containing protein 5	Nucleus	C	4
ZNF462	Zinc finger protein 462	Nucleus	D	3
SIRT6	NAD-dependent protein deacetylase sirtuin-6	Nucleus	D	2
MAEA	E3 ubiquitin-protein transferase MAEA	Nucleus	D	1
RERE	Arginine-glutamic acid dipeptide repeats protein	Nucleus	D	1
ZNF142	Zinc finger protein 142	Nucleus	D	1
**RPL5**	**60S ribosomal protein L5**	**Cytoplasm/nucleus**	**C**	**4**
NF1	Neurofibromin	Cytoplasm/nucleus	D	1
QSCN6 / QSOX1	Sulfhydryl oxidase 1	Cytoplasm	C	4
MMP15	Matrix metalloproteinase-15	Membrane	A	21
CLIP4	CAP-Gly domain-containing linker protein 4	Unknown	D	4
**(B) Lmo2027 Y2H screen (this study)**				
DNAJB5	DnaJ homolog subfamily B member 5	Nucleus	C	2
PDS5	Sister chromatid cohesion protein PDS5 homolog B	Nucleus	D	1
CAPN7	Calpain 7	Cytoplasm/Nucleus	A	6
SMURF1	E3 ubiquitin-protein ligase SMURF1	Cytoplasm/Nucleus	D	1
**(C) InlP Y2H screen by Farrala et al. (2018)**^34^				
**RBM5**	**RNA-binding protein 5**	**Nucleus**	**A**	**14**
TSHZ1	Teashirt homolog 1	Nucleus	A	12
AEBP1	Adipocyte enhancer-binding protein 1	Nucleus	A	7
NAB2	NGFI-A-binding protein 2	Nucleus	A	6
THAP3	THAP domain-containing protein 3	Nucleus	A	6
NAB1	NGFI-A-binding protein 1	Nucleus	A	4
**DNMT3A**	**DNA (cytosine-5)-methyltransferase 3A**	**Nucleus**	**B**	**5**
SAFB	Scaffold attachment factor B1	Nucleus	B	5
ZNF653	Zinc finger protein 653	Nucleus	B	5
SP3	Transcription factor Sp3	Nucleus	B	3
ZNF711	Zinc finger protein 711	Nucleus	C	3
CDC27	Cell division cycle protein 27 homolog	Nucleus	C	2
ZNF283	Zinc finger protein 283	Nucleus	C	2
**RPL5**	**60S ribosomal protein L5**	**Cytoplasm/nucleus**	**A**	**9**
EXOSC3	Exosome complex component RRP40	Cytoplasm/nucleus	B	4
RPL7A	60S ribosomal protein L7a	Cytoplasm/nucleus	B	4
PHF23	PHD finger protein 23	Cytoplasm/nucleus	C	2
RPS8	40S ribosomal protein S8	Cytoplasm	A	26
COLGALT1	Procollagen galactosyltransferase 1	Cytoplasm	B	3
EPS8L2	Epidermal growth factor receptor kinase substrate 8-like protein 2	Cytoplasm	C	2
AF6	Afadin	Membrane	B	3

List of protein partners identified in the present study (**A,B**) or by Faralla et al. (2018)^34^ (**C**). Ranking is based on the subcellular localization of the prey, then on the predicted biological score (score: A: very high confidence; B: high confidence; C: good confidence. D: Moderate confidence), and then on the number of clones. InlP partners found in both A and C are in bold.

**Figure 3:**
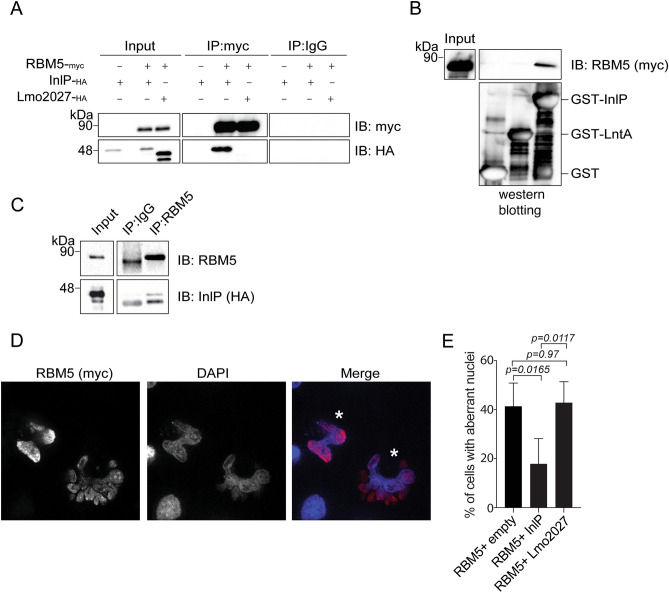
InlP targets RBM5. (**A**) Nuclear extracts from HEK293-FT cells co-expressing RBM5-_Myc_ and InlP-_HA_ or Lmo2027-_HA_ were used in immunoprecipitations (IP) assays with Myc antibodies or IgG control and analyzed by immunoblots with Myc or HA antibodies. (**B**) Nuclear extracts from HEK293-FT cells transfected with RBM5-_Myc_ were incubated with purified GST, GST-LntA, or GST-InlP. Immunoblots of inputs and eluted fractions were probed with antibodies against Myc or GST. A comassie gel staining of the same fractions is shown below. (**C**) Immunoprecipitation of endogenous RBM5 from HeLa cell transfected with InlP-_HA_. Immunoblots of inputs and eluted fractions were probed with antibodies against endogenous RBM5 and HA. (**D**) Representative image of the altered nuclear morphology of HeLa cells transfected with RBM5-_Myc_ **(**asterisks indicate aberrant nuclei). (**E**) Percentage of cells showing aberrant nuclei after transfection of RBM5-_Myc_ or co-transfection of RBM5-_Myc_ and InlP-_HA_ or RBM5-_Myc_ and Lmo2027-_HA_. Data are mean ± SD from at least three independent biological replicates. Statistical significance determined by ANOVA.

We next sought to validate the interaction between InlP and RBM5 by immunoprecipitation. Transfected InlP-_HA_ co-immunoprecipitated with co-expressed RBM5-_Myc_, while Lmo2027-_HA_ did not (Fig. 3A). Additionally, in pull-down assays, recombinant GST-InlP, expressed and purified from *E. coli*, pulled-down overexpressed RBM5-_Myc_ from nuclear extracts, whereas GST or another *L. monocytogenes* nucleomodulin (GST-LntA) did not, thus confirming the specific interaction between InlP and RBM5 (Fig. 3B). Finally, endogenous RBM5 pulled down overexpressed InlP-_HA_ from cell lysates (Fig. 3C).

The RBM5 protein is a tumor suppressor that stimulates apoptosis when overexpressed^35^. We therefore tested the functional role of the InlP-RBM5 interaction by evaluating the impact of InlP on the pro-apoptotic effect of RBM5. In HeLa cells, RBM5-induced apoptosis is easily visualized by the presence of nuclei with highly altered morphologies (i.e., condensed or fragmented nuclei) identifiable by Hoechst staining^35^ (Fig. 3D). We used this assay in HeLa cells co-transfected with RBM5 and either InlP or Lmo2027. In RBM5-overexpressing cells, the number of aberrantly shaped nuclei increased by approximately 40% compared to cells transfected with the empty vector (Fig. 3E). In contrast, when InlP was co-expressed with RBM5, the number of aberrantly shaped nuclei was greatly reduced (to ~ 13%), an effect not observed in the presence of Lmo2027 (Fig. 3E).

Altogether, these results identify the splicing factor RBM5 as a host binding partner for InlP and strongly suggest that InlP acts as an inhibitor of RBM5 in the context of the RBM5-induced cell death.

### The OCRE domain of RBM5 mediates the functional interaction with InlP

**Figure 4:**
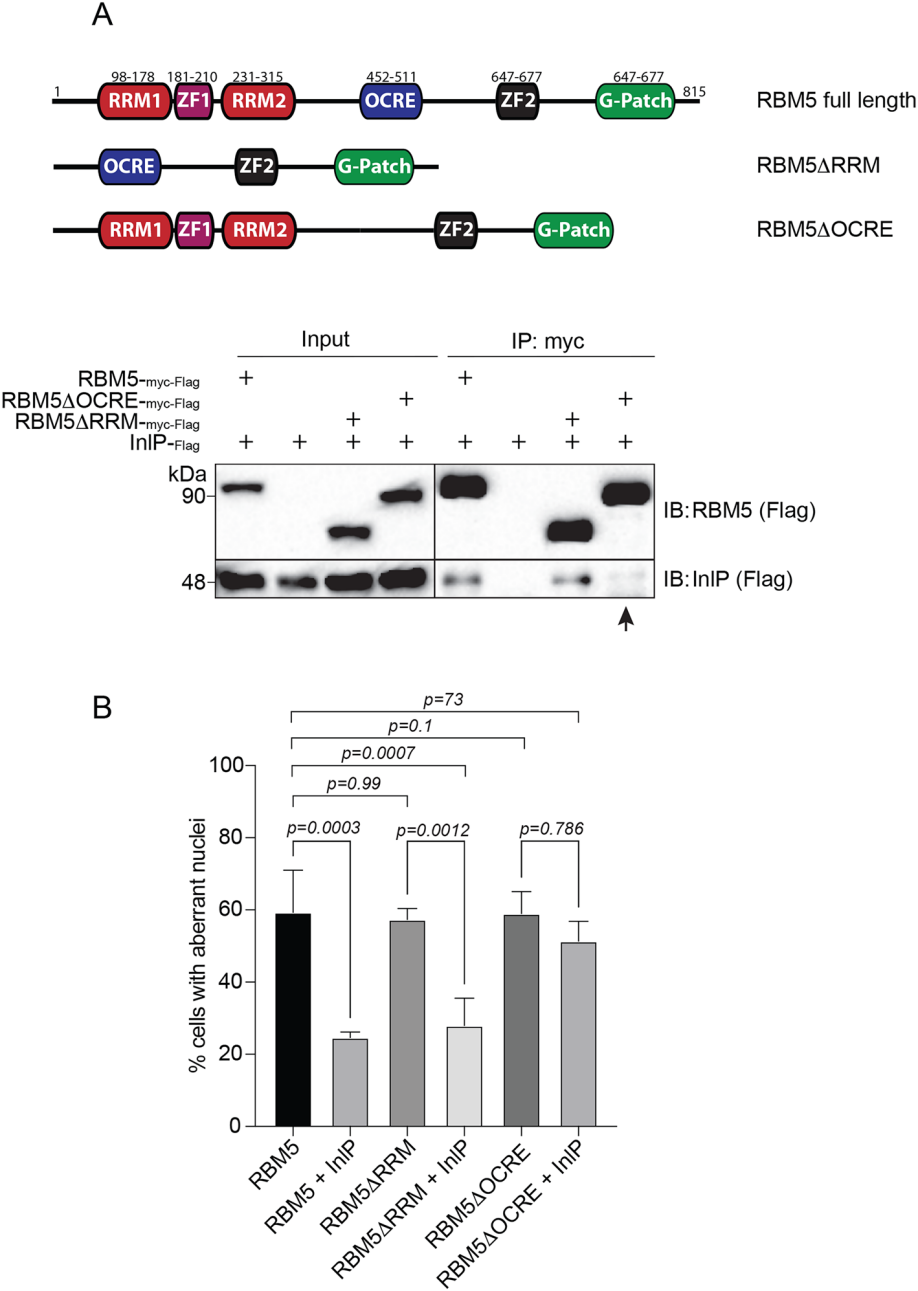
The OCRE domain of RBM5 is required for the functional interaction with InlP. (**A**) Top, schematic representation of RMB5 full length and mutants used in immunoprecipitation experiments. Bottom, Myc immunoprecipitation (IP) of RBM5-_Myc-FLAG_ or RBM5 mutants (RBM5∆OCRE_Myc-FLAG_; RBM5∆RRM_Myc-FLAG_) in HEK293-FT cells co-expressing or not InlP-_HA-FLAG_. The FLAG antibody was used to detect RBM5 and InlP on the same immunoblot for comparative purposes. The arrow points to the impaired interaction between RBM5∆OCRE and InlP. (**B**) Percentage of cells showing aberrant nuclei after transfection of RBM5, RBM5∆RRM, RBM5∆OCRE, in presence or absence of InlP. Data are mean ± SD from at least three independent biological replicates. Statistical significance determined by ANOVA.

We then sought to determine which region of RBM5 was required for the inhibitory effect of InlP. RBM5 is a multidomain protein of 92 kDa belonging to the family of RNA-binding motif proteins. Among the different domains, RBM5 has two RNA-recognition motifs (RRM1 and RRM2), which bind RNAs, and an Octamer Repeat of Aromatic Residues (OCRE) domain, which mediates interaction with small nuclear ribonucleoproteins (snRNPs) and components of the splicing machinery^36,41^. To investigate whether either of these two domains might be involved in interaction with InlP, we generated deletion mutants of each domain and tested them in co-immunoprecipitation assays with InlP (Fig. 4A). While RBM5 ∆RRM1-2 still bound InlP, the ∆OCRE mutant showed a strongly impaired interaction (Fig. 4A). Thus the OCRE domain mediates the interaction with InlP.

Regarding RMB5-induced cell death, the RBM5 ∆RRM1-2 and ∆OCRE mutants were as effective as RBM5 in inducing cell death, with 60% of transfected cells showing aberrant nuclei (Fig. 4B). However, while InlP could efficiently counteract cell death in both full length RBM5 and RBM5 ∆RRM1-2-expressing cells, this inhibitory effect was strongly reduced in RBM5 ∆OCRE-expressing cells (Fig. 4B). These results indicate that the inhibitory effect of InlP on RBM5-dependent cell death requires the interaction between InlP and the OCRE domain of RBM5. However, we cannot rule out that the overexpression of the ∆OCRE mutants might have some toxic effect.

### InlP stimulates the formation of RBM5-dependent nuclear granules

**Figure 5:**
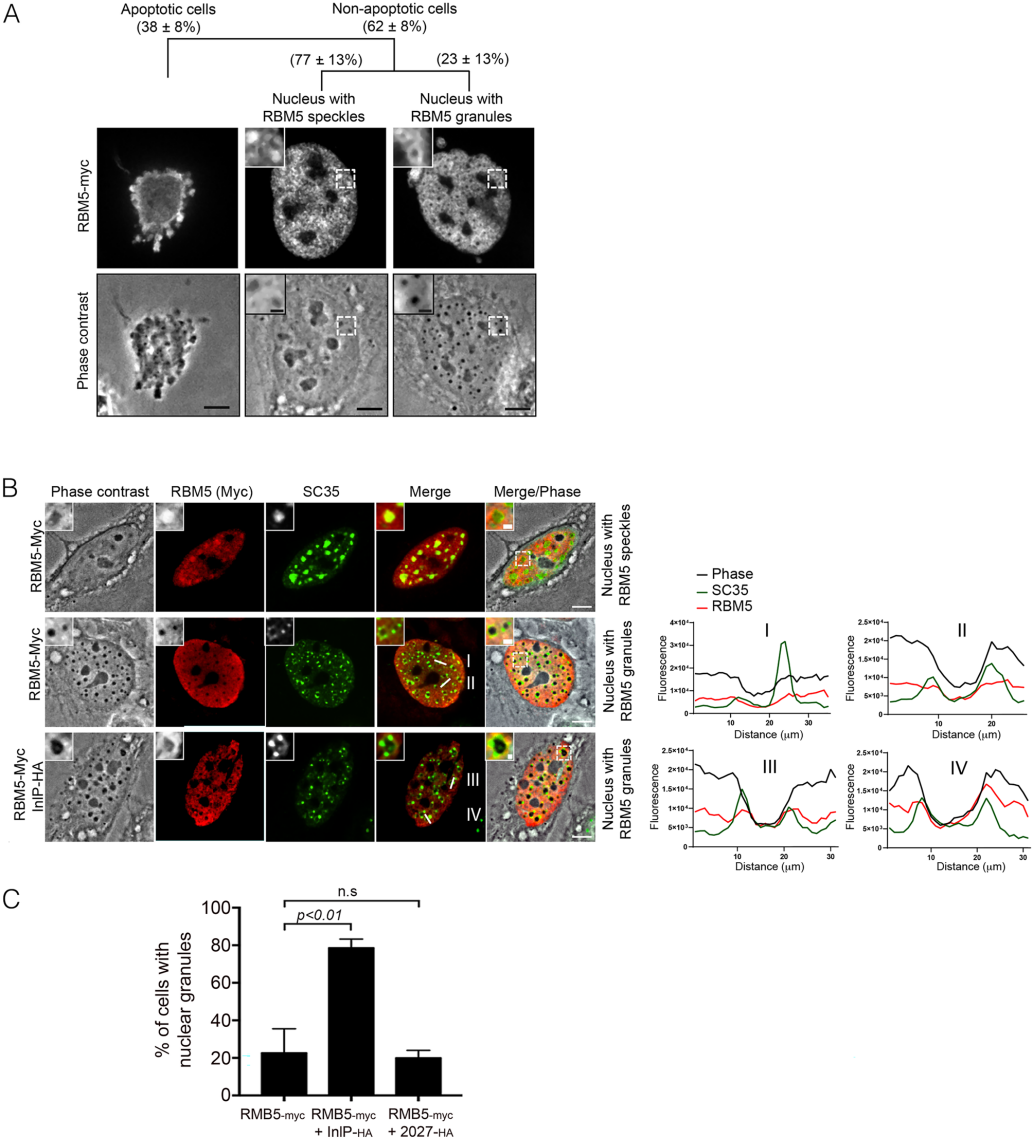
InlP stimulates formation of RBM5-associated nuclear granules. (**A**) Representative micrographs of the three subtypes of RBM5-_Myc_-expressing cells, with the RBM5-_Myc_ immunostaining on top and the phase-contrast image below (bars: 10 µm). A higher magnification of each squared region is shown in the upper left corner (bars: 2 µM). The percentage of each cell subtype is indicated as a mean ± SD of 3 independent experiments, with n = 30 cells per replicate. (**B**) Left, representative phase contrast and immunofluorescence confocal microscopy images of transfected RBM5-_Myc_ and SC35, in presence or absence of InlP-_HA_. Three subtypes of RBM5-expressing HeLa cells are shown: a cell with a nucleus containing RBM5-speckles (first row); a cell with a nucleus containing RBM5 granules (second row); a cell with a nucleus containing RBM5 granules in presence of InlP (third row). Scale bars: 10 µm. A higher magnification of each boxed region in the Merge/Phase image is shown in the upper left corner (Scale bars: 2 µm); Right, localization profile of RBM5 and SC35 assessed with a line scan (white lines in the merged images) whose fluorescence intensity is plotted in red for RBM5, in green for SC35 and in black for RBM5 granules; faint co-localization between RBM5 and SC35 is observed in I and II, while a more extensive colocalization is measured in III and IV. (**C**) Nuclei with RBM5 granules were scored in HeLa cells transfected RBM5-_Myc_ or co-transfected with RBM5-_Myc_ and InlP-_HA_ or Lmo2027-_HA_. Histograms represent percentage of each category as mean ± SD of 3 independent experiments. Statistical significance determined by ANOVA.

The interaction between InlP and RBM5 and the observation that InlP inhibited RBM5-induced cell death prompted us to carefully assess the localization of RBM5 in the nucleus of non-apoptotic cells, and whether InlP could have any impact. As antibodies to endogenous RBM5 weakly detected RBM5 in immunofluorescence assays, we analyzed RBM5 localization in cells ectopically expressing RBM5-_Myc_ and used an anti-Myc antibody for detection. By quantifying cells with aberrant nuclei, we estimated the non-apoptotic cell population to be ~ 60% of RBM5-_Myc_-positive cells (Fig. 5A), and noted that they divided into two types of populations. In ~ 80% of them, RBM5 was concentrated in nuclear bodies (Fig. 5A, column 2), as previously reported in cells expressing GFP-tagged RBM5^34^, where they were termed “RBM5 speckles”. In contrast, in the remaining ~ 20% population, phase contrast microscopy highlighted nuclei containing several circular dense granules that were darker than speckles and did not co-localize with nucleoli (Fig. 5A, column 3, Fig. S4). These granules were not stained by DAPI, indicating that they were not areas of compact chromatin. In addition, we observed instances of RBM5 surrounding these granules. We named these structures “RBM5 granules”. Furthermore, as previously shown in cells expressing RBM5-GFP, RBM5-_Myc_ co-localized with the splicing protein SC35 in RBM5 speckles (Fig. 5B, row 1). However, the distribution of SC35 was profoundly altered in nuclei with RBM5 granules: instead of being organized in a speckle pattern (Fig. 5B, row 1), SC35 appeared to be concentrated as small dots at the edge of RBM5 granules (Fig. 5B, row 2).

When InlP was co-expressed with RBM5, not only were these SC35 punctate-rich granules observed in nuclei, but the RBM5 rings surrounding these structures were more visible (Fig. 5B, row 3). In addition, InlP significantly stimulated the formation of these granules, from 20 to 80% of nuclei harboring such structures (Fig. 5C). Importantly, co-expression of the InlP paralog Lmo2027 with RBM5 had, in contrast, no effect (Fig. 5C). Together, these results reveal that RBM5 can induce the formation of a new type of nuclear bodies, which are granules enclosed by SC35, and that the bacterial factor InlP stimulates both the formation as well the recruitment of RBM5 around these structures.

We next investigated whether the interaction between InlP and RBM5 was necessary for InlP to stimulate RBM5 granules, ruling out possible indirect effects. For this purpose, we used the OCRE mutant of RBM5 unable to interact with InlP. When overexpressed in cells, RBM5 ∆OCRE induced the formation of nuclear granules as efficiently as the full length RBM5 protein. However, the co-overexpression of InlP stimulated the formation of nuclear condensate only in RBM5-expressing cells and had a very minor effect in RBM5 ∆OCRE-expressing cells (Fig. S5). These results suggest that the interaction between InlP and RBM5 contribute to the formation of RBM5 condensates.

### InlP affects the structure of nuclear RBM5 granules

**Figure 6:**
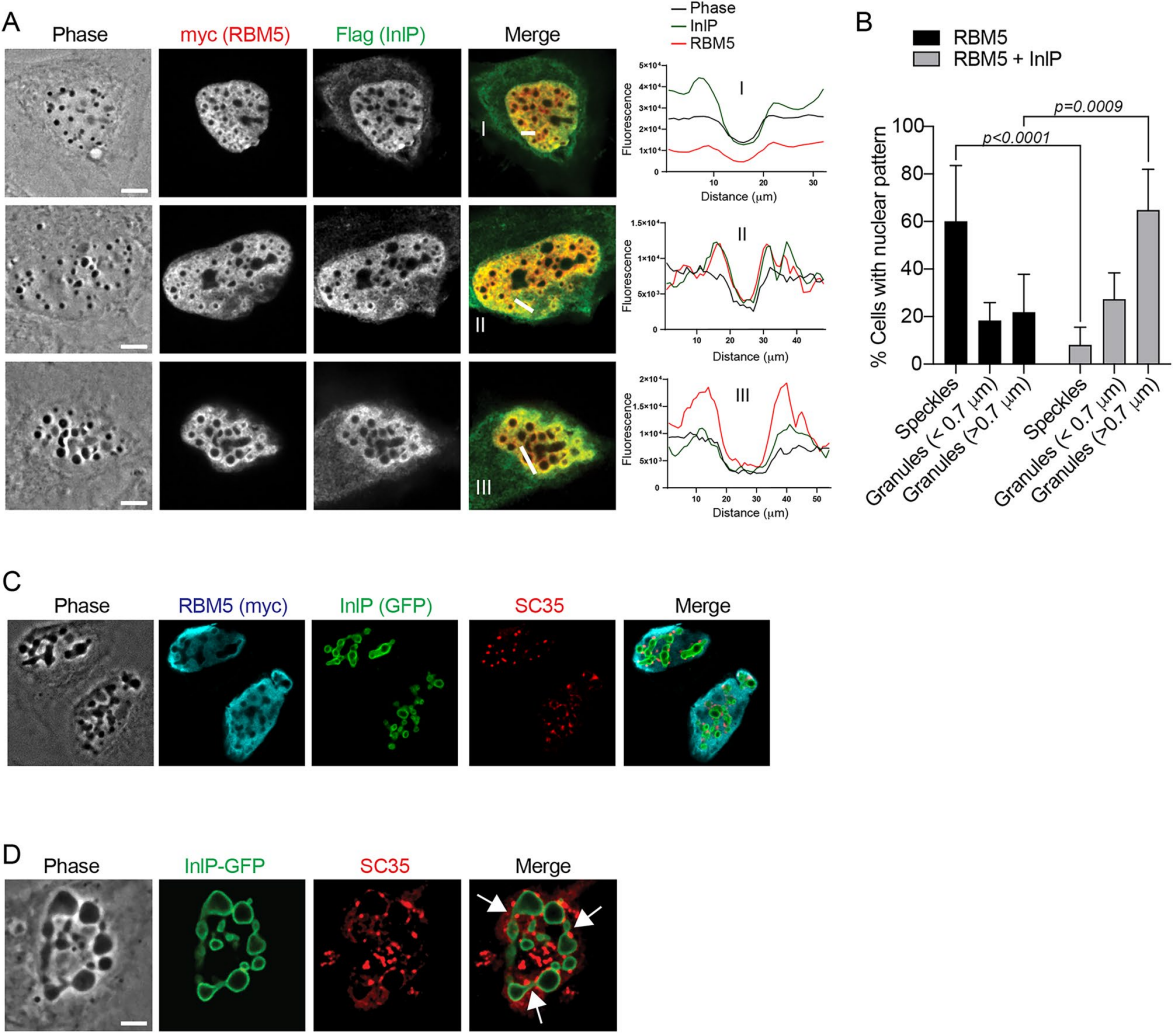
Co-expression of RBM5 and InlP stimulates the redistribution of SC35-speckles at large deformed nuclear condensates. (**A**) Left, representative phase contrast and immunofluorescence confocal microscopy images of HeLa cells co-transfected with RBM5-_Myc_ and InlP-_HA_ (three examples are shown, with RBM5 condensates increasing in size); right, localization profile of RBM5 and InlP assessed with a line scan (white lines in the merged images) whose fluorescence intensity is plotted in red for RBM5, in green for InlP and in black for RBM5 granules; co-localization between RBM5 and SC35 is observed in I II, and III. (**B**) Nuclei with RBM5 speckles or RBM5 granules were scored in RBM5-expressing HeLa cells, in absence (black bars) or presence (grey bars) of InlP. Histograms represent percentage of each category as mean ± SD of 3 independent experiments. Statistical significance determined by ANOVA. (**C,D**) Representative phase contrast and immunofluorescence confocal microscopy images of HeLa cells co-expressing RBM5-_Myc_ and GFP-InlP, immunolabeled with SC35 and RBM5 antibodies (A) or SC35 antibody (B). A nucleus with RBM5-condensates formed in presence of GFP-InlP is shown at higher magnification in (D). Arrows point to sites that show potential events of coalescence or fission. Scale bars: 10 µm.

We noticed that in the presence of InlP, RBM5 granules were often larger and their number per nucleus decreased as the size of these structures increased (Fig. 6A,B). In order to simultaneously visualize RBM5, InlP and SC35 in the nucleus, we generated a vector expressing InlP fused to GFP (GFP-InlP). In nuclei co-expressing RBM5-_Myc_ and GFP-InlP, RBM5 granules appeared large and clearly delimited by the GFP-InlP fluorescence signal and the dotted staining of S35 (Fig. 6C). In addition, these large GFP-InlP positive granules often showed deformations reminiscent of fusion and/or fission events (Fig. 6D, see arrows). These results suggest that the bacterial factor InlP not only stimulates the formation but may affect the dynamics of RBM5-induced nuclear granules. InlP could promote the aggregation of RBM5 and RBM5-associated splicing factors, thereby impacting their sub-nuclear compartmentalization.

## Discussion

Deciphering the mechanisms by which pathogens’ effectors target and manipulate host cell components is critical to the understanding of infectious diseases and can lead to a better characterization of fundamental processes in cell biology. However, some bacterial effectors are not produced in vitro, making difficult to decipher their molecular mechanism of action. Here, we report that InlP is one of the *L. monocytogenes* virulence factors that are not produced by bacteria grown in a BHI-rich medium, as described previously for InlJ^21^, InlE^20^, InlK^24^, LntA^9^ and OrfX^13^. We provide several pieces of evidence that InlP is an additional nucleomodulin^8^ of *L. monocytogenes*: InlP localizes to the nucleus and changes the shape of splicing speckles; InlP binds the splicing factor RBM5 and stimulates the formation of RBM5-associated nuclear granules. Additionally, InlP inhibits RBM5-mediated cell death. Our work uncovers the first *L. monocytogenes* effector targeting a component of the host splicing machinery.

We particularly found that InlP affects the spatial distribution of splicing factors. Our study first shows that the presence of InlP in the host cell nucleus alters the architecture of SC35-associated splicing speckles. Splicing speckles as other nuclear bodies, such as the nucleoli (which drive ribosomal RNA synthesis), Cajal bodies (which catalyze the biogenesis of small ribonucleoproteins), and PML bodies (which store various nuclear compounds)^39^, are MLO. They behave like viscous droplets dynamically formed by a mechanism of liquid–liquid phase separation^40,42,43^ or “condensation”, thus also being referred to as biomolecular condensates^44,45^. Their assembly depends on the critical concentration of certain protein domains involved in the formation of heterogeneous networks, which trigger phase separation. We propose that InlP could change the morphology of splicing speckles by interacting and redistributing components stored in these structures, although we cannot rule out other mechanisms for such an effect^46^. Our study also reports that InlP interacts with the splicing factor RBM5, which contains RRM (“RNA recognition motif”) and RS (“Arg-Ser low complexity repeats”) domains known to play a role in the condensation phenomenon^42^. In RBM5-overexpressing cells, InlP induces the massive appearance of RBM5-associated nuclear granules, which could result from phase separation induced when a critical concentration of RBM5 is reached. In addition, the speckle protein SC35 relocates at the edge of RBM5-associated nuclear granules in a dotted-like pattern. The relocation of SC35 could also contribute to the formation of these bodies, via the RRM and RS domains of SC35. Such an organization has been described for paraspeckles, with the protein RBM14 playing the role of interface between the shell and the core of a paraspeckle^39^. One model suggests that nuclear bodies are structured into distinct zones, including a peripheral region termed “shell”, regulating the interface with the rest of the nucleoplasm. By locally concentrating or aggregating RBM5 and SC35 ligands, InlP could stimulate the process of phase separation and coalescence of RBM5 condensates. We propose that this phenomenon, which is visible on the micrometer scale following the overexpression of RBM5 and InlP, could take place at a smaller scale under physiological conditions (i.e., infection by *L. monocytogenes *in vivo). Therefore, the exact role of InlP during *L. monocytogenes* infection in vivo remains to be addressed.

While intracellular bacterial pathogens are well known to manipulate organelles delimited by membranes in the cytoplasm, their impact on MLO has only begun to emerge in recent years. A few examples illustrate these effects. For cytosolic MLO, *Salmonella* disassembles RNA and protein aggregates, known as P-bodies, involved in post-transcriptional regulation^47^; *Shigella* inhibits the aggregation of stress granules^48^; *Shigella, Salmonella* and *L. monocytogenes* induce the aggregation of small ribonucleoproteins-containing bodies, through metabolic stress^49^. For nuclear MLO, the LLO toxin from *L. monocytogenes* triggers oxidative stress, a signal that subsequently induces the aggregation of PML bodies and their association with the nuclear matrix^50^. However, regarding a direct effect, to our knowledge only the nucleolus is so far known to be targeted by nucleomodulins: *Legionella* and *Burkholderia* spp. secrete effectors that modify the chromatin of rDNA in the nucleolus^51^; *E. coli* effector EspF causes the loss of nucleolin, the most abundant nucleolar protein^52^; *Coxiella burnetii* effector NopA localizes to the nucleolus and perturbs nucleocytoplasmic transport^53^. InlP adds another activity to the bacterial pathogen “toolbox” by acting on the spatial distribution of splicing factors SC35 and RBM5 and possibly other nuclear RBPs.

In future research, it will be important to address whether InlP can modulate the RBM5-dependent splicing. This will require a prior identification of the RNA targets whose alternative splicing is affected by RBM5 during *L. monocytogenes* infection. Alternative transcript splicing is a complex process by which exons of a pre-mRNA molecule are differentially assembled after introns are removed. The target pre-mRNAs depend on the nature of the cells and tissues and on physiological or pathological signals. Bacterial pathogens can induce alteration of the host cell splicing landscape, but the mechanisms described to date are likely to be indirect^54^. To our knowledge, only one study has identified a bacterial effector that can directly target the splicing machinery. This effector, IpaH9.8 from *Shigella flexnerii* is, like InlP, a LRR protein^55^. IpaH9.8 binds to the splicing factor U2AF35, but its activity on splicing of specific transcripts has not been evaluated in the context of infection but only by using model minigenes.

InlP was initially described for a role on the dynamics of intercellular junctions in epithelial cells via its interaction with Afadin^31^. InlP thus shares with other bacterial LRR-containing proteins the ability to target various host proteins at spatially distinct locations. For example, *L. monocytogenes* InlC acts on the dynamics of intercellular junctions by targeting TUBA^25,26^ and on the innate immune responses by targeting IKKα^27,28^. Similarly, several LRR-containing effectors from Gram-negative pathogenic bacteria, such as IpaH9.8, have multiple ligands and functions^18^. The fact that InlP acts on the assembly or dynamics of nuclear bodies suggests that it may control the storage of its target proteins in nuclear microenvironments, thereby altering their cellular functions. Consistent with this hypothesis, our results show that InlP inhibits the pro-apoptotic function of RBM5. The secretion of an anti-apoptotic effector could be beneficial to intracellular *L. monocytogenes* by promoting the survival of their niche, particularly in the context of a long-term infection. Likewise, other pathogenic bacteria produce nucleomodulins with anti-apoptotic activities, such as the *C. burnetii* effector AnkG^56^ and *Brucella abortus* effector BspJ^8^. As for InlP, the nuclear localization of these proteins was established by ectopic expression in host cells. The nuclear function of InlP during *L. monocytogenes* infection remains to be precisely established.

The molecular signals that trigger *inlP* gene expression during infection are unknown. Neither the work of Faralla et al.^30,31^ nor our work elucidated the conditions required for *inlP* activation*,* at the transcriptional or post-transcriptional level, by intracellular *L. monocytogenes.* It is possible that *inlP* transcription, as well as its secretion/stability require cofactors that are not present under these particular experimental conditions. Further studies are therefore required to identify the signal(s) triggering the expression of InlP. Of note, a phylogenomic approach focused on the co-evolution of *L.* *monocytogenes* small non-coding RNAs (sRNA) and their target mRNAs has identified a significant co-evolution of a sRNA (rli133) with three nucleomodulin-coding genes that are not expressed in vitro: *lntA, orfX* and *inlP*^57^. This observation opens the possibility that bacteria produce these nucleomodulins at the same stage of infection to control the expression of host genes by different mechanisms. Regarding the nuclear-interacting partner of InlP, RBM5, it is expressed in both placenta and brain (data from the Human Protein Atlas database), two organs targeted by *L. monocytogenes*. This opens up interesting research opportunities to study the function of InlP and RBM5, as well as of their RNA targets, in maternal-neonatal and neuromeningeal listeriosis. In this regard, it is worth mentioning that neuronal RBM5 is upregulated in certain brain regions after injury and appears to control the splicing of specific brain transcripts^36^, which may be important in response to bacterial infection.

## Materials and methods

### Human cell lines, bacterial strains and plasmids

Human HeLa cells (ATCC CCL2), HEK293-FT (Invitrogen), JEG-3 (ATCC HBT-36), HepG2 (ATCC HB-8065) and LoVo cells (ATCC CCL229) were cultured at 37 °C under an atmosphere of 5% CO_2_, in media recommended by ATCC (Manassas, VA). The culture media (GIBCO) was supplemented with 10% Fetal Calf Serum (FCS) (Sigma). *L. monocytogenes* and *Escherichia coli* strains were grown in Brain–Heart Infusion (BHI) medium or Luria–Bertani (LB) medium (Difco, BD), respectively, with antibiotics in the presence of plasmids. Bacterial strains and plasmids are described in the Supplementary Information.

### Yeast two-hybrid (Y2H) screening

Y2H was performed as described previously^9,11^. Briefly, pB27-*inlP* or pB27-*lmo2027* was transformed in yeast L40DGAL4 and screening was performed by Hybrigenics on the human placenta cDNA library, with 105 million (InlP screen) or 126 million (Lmo2027 screen) interactions tested.

### Antibodies and reagents

The InlP polyclonal antibody was obtained after injection of an immunogenic peptide (amino acids 365 to 379, LDVSYNHNYATGGVC) in rabbits and subsequent affinity purification. The InlC polyclonal antibody is described in^27^. The other primary antibodies are polyclonal antibodies against RBM5 (Sigma, HPA017335 for IF and Bethyl, A302-228A for IP), PML (Abcam, sc-966), anticoilin (Proteintech, 10967-1-AP), anti-nucleolin (Santa cruz, sc-13057) and monoclonal antibodies against tubulin (hybridoma E7), SC35 (AbCam, ab11826), FLAG-M2 (Sigma-Aldrich, F1804), Myc (9E10, Santa Cruz Biotechnology, sc-40), HA (6E2, Cell Signaling technology #2367), V5 (R960-2, Invitrogen). The immunoprecipitation control antibodies are IgG mouse (Santa-Cruz, sc-2025) and IgG rabbit (Santa-Cruz sc-2027). The secondary antibodies are coupled to Alexa-488 (Life technologies) or Cy3 or Cy5 (Jackson ImmunoResearch). DAPI and Hoechst are from Roche Applied Sciences and Thermo Fisher Scientific, respectively. Lipofectamine LTX Max is from Invitrogen.

### Assays for InlP secretion in BHI medium or during infection of JEG-3 cells

One mL of each bacterial culture was centrifuged 5 min at 2000 × *g*. The supernatant was filtered through 0.22 µm filters and the proteins were precipitated by addition of TCA (Trichloroacetic acid) at 16% final for 45 min on ice, followed by a 45 min centrifugation at 15000 *g* at 4 °C. The supernatant proteins were then rinsed twice with cold acetone and recovered after 15 min centrifugation. The supernatant protein pellet was dried and then resuspended in 200 µL Laemmli deposition buffer. The bacterial pellet was washed twice with PBS (Phosphate Buffered Saline) before being resuspended in 200 µL Laemmli buffer. Proteins were analysed by western blot. To study InlP secretion during infection, JEG-3 cells were seeded into 6-well plates two days prior to infection. On the day prior to infection, the bacterial strains were cultured at 37 °C in liquid BHI with agitation. One mL of each overnight culture was centrifuged 5 min at 2000 × *g*, the pellet was washed twice in PBS 1X and diluted 10,000 times in MEM culture medium. The cells were washed with MEM before adding 2 mL of the bacterial suspension to achieve a multiplicity of infection of about 50. Plates were centrifuged 2 min at 300 × *g* to synchronize infection and then incubated at 37 °C for one hour. The medium was then replaced by complete MEM medium, with the addition of 25 µg/mL gentamicin to kill extracellular bacteria, for 4 to 8 h. The cells were gently lysed in hypotonic buffer using 20 passages in wheaton dounce (model 2-5 mL), then 91 µL sucrose buffer was added and the mixture was centrifuged 5 min at 500 × *g*. 10% of the volume of the recovered supernatant was then removed to be equilibrated to 60 µL with SDB buffer. After addition of the final Laemmli 1X deposition buffer, the preparations are used for western blot analysis.

### GST-pulldowns and immunoprecipitations assays

GST-InlP, GST-LntA or GST recombinant proteins were purified from *E. coli* BL21(DE3) bacteria transformed with the plasmids pGEX4T1-InlP, pGEX4T1-LntA^10^ or pGEX4T1. 50 mL of cultures at DO600 = 0.6 were induced 3 h at 30 °C with 0.5 mM IPTG. The bacterial pellets were lysed by sonication in PBS supplemented with a full cocktail of protease inhibitor (Sigma) and bacterial lysates were incubated with 50 μl of a 50% suspension of magnetic glutathione beads (Amersham, Biosciences), for 1.5 h at 4 °C on a wheel, then washed twice with wash buffer (20 mM Tris pH = 7.65; 150 mM NaCl; 0.5% IGEPAL; 2.5% Glycerol; 0.5 mM EDTA; 34 mM sucrose; 0.6 mM DTT). The amount of GST fusion protein was estimated by Coomassie blue staining to normalize the amounts used in the pull-down experiments. Pre- and post-bead lysed bacterial extracts were also collected for evaluation by Coomassie blue staining. Nuclear extracts were prepared as described above. The nuclear (soluble + chromatin) and cytoplasmic extracts were stored at − 80 °C. 50 μl of purified nuclear extracts were mixed with appropriate amounts of GST magnetic beads, supplemented with protease inhibitors and incubated overnight at 4 °C. The beads were then washed five times with the same wash buffer and 50 μl Laemmli 2X deposition blue was added and the mixture was denatured for 10 min at 100 °C. 15 μl supernatant was loaded into SDS-PAGE gel.

Co-immunoprecipitations of RBM5-_Myc_ with InlP-_HA_ or Lmo2027-_HA_ were performed from nuclear extracts from HEK293-FT cells co-transfected or not with the indicated vectors, according to the protocol described in^12^, using magnetic beads conjugated with an anti-myc antibody. Nuclear fractions were prepared as previously described^9^. Immunoprecipitation of endogenous RBM5, or of transfected RBM5-_Myc-FLAG_, RBM5∆RRM1-2-_Myc-FLAG_ and RBM5∆OCRE-_Myc-FLAG_ proteins, with or without InlP-_HA-FLAG_, was performed from HeLa total cell lysate. Briefly, HeLa cells in 10-cm2 dishes were co-transfected with the indicated vectors using Lipofectamine LTX Invitrogen. 24 h after transfection, cells were washed twice with PBS and lysed using 1 mL of lysis buffer per dish (50 mM TrisHCl pH 7.5, 150 mM NaCl, 1% Igepal CA-630, 1% SDS, 5% glycerol, 1 mM DTT supplemented with protease inhibitors mixture) and incubated for 30 min with shaking, 4 °C. Cell lysate was then clarified (13,000 g, 10 min, 4 °C) and assayed for protein concentration (Bradford). 0.5–1 mg of total lysate was incubated with 3 μg of the antibodies (endogenous RBM5 or Myc antibodies) and 50 μl of protein G or protein A magnetic beads (Invitrogen), overnight, 4 °C. Beads were recovered and washed three times in lysis buffer. Proteins were eluted by boiling beads in 50 μl Laemmli buffer with 100 mM DTT. Samples were then subjected to immunoblotting via transfer onto a 0.45-μm nitrocellulose membrane and incubation with the indicated antibody. Western blotting procedures are detailed in the Supplementary Information.

### Immunofluorescence, microscopy and image analysis

HeLa cells were seeded into 6-well or 24-well plates containing sterile glass coverslip and cultured up to 70% confluence and transfected with a mixture of Lipofectamine LTX (Invitrogen) and plasmid DNA. 24 h later, the cells were washed with PBS and then fixed for 30 min via the addition of 4% PFA (Paraformaldehyde) in PBS. After two PBS washes, cells were permeabilized 4 min with 0.4% PBS-Triton X-100, blocked with 2% PBS-BSA (Bovine Serum Albumin), and incubated in 2% PBS-BSA containing the primary antibody for one hour. The slides were rinsed three times with PBS, incubated for one hour in PBS-BSA 2%, containing the secondary antibody coupled to a fluorochrome, as well as DAPI or Hoechst, washed in PBS and then milli-Q water. After mounting on slides with mounting liquid (Interchim, FluoroMount-G), the slides were observed and analyzed with an epifluorescence microscope (Carl Zeiss, Axiovert 135, AxioObserver.Z1), or a confocal microscope (Yokogawa CSU-X1 confocal spinning disk microscope). The images were acquired with a 40 ×, 63 × or 100 × non-immersion objective, and the images were processed with Zen (Carl Zeiss), MetaMorph (Universal Imaging) or ImageJ softwares. Image quantification is detailed in the Supplementary Information. For time-lapse microscopy, HeLa cells were seeded in 35 mm glass-bottom dish (MatTek) and cultivated in DMEM 10% FCS without phenol red medium. Images were acquired on Zeiss AxioObserver.Z1 equipped with an incubation chamber (37 °C and 5% CO_2_) and a non-immersion 40 × objective.

### Statistical analysis

Data were expressed as means ± standard deviation (SD) and analyzed using a student's t-test or an ANOVA. Differences in means were considered statistically significant at *p* < 0.05. Calculations were performed with GraphPad Prism or Excel software.

## Supplementary Information

Supplementary Information.

Supplementary Figures.

## Data Availability

All data generated or analyzed during this study are either included in this published article and its supplementary information files, or are available from the corresponding author on reasonable request.
