# Supplementary material for: A bacterial virulence factor interacts with the splicing factor RBM5 and stimulates formation of nuclear RBM5 granules
Source: Sci Rep. 2022 Dec 19;12:21961. doi: 10.1038/s41598-022-26037-w (PMC9763339; doi:10.1038/s41598-022-26037-w)
Supplement: Supplementary file 1 — Supplementary Information. [file 41598_2022_26037_MOESM1_ESM.docx]

**Supplementary Informations**

**A bacterial virulence factor interacts with the splicing factor RBM5 and stimulates formation of nuclear RBM5 granules**

Renaud Pourpre^1$^, Goran Lakisic^1$^, Emma Desgranges^1^, Pascale Cossart^2^, Alessandro Pagliuso^1*^ and Hélène Bierne^1,3^

^1^ Université Paris-Saclay, INRAE, AgroParisTech, Micalis Institute, EpiMic Lab, Jouy-en-Josas, France.

^2^ Institut Pasteur, Paris, France.

^3^ Deceased during the revision process

$ These two authors have equal contribution to this work.

* corresponding author:

[alessandro.pagliuso@inrae.fr](mailto:alessandro.pagliuso@inrae.fr)

**1. Supplementary Materials and Methods**

**1.1. Bacterial strains**

The bacterial strains used in this work are listed below.

| Strain | Description | Number | Selection  (µm/mL) | Reference |
| --- | --- | --- | --- | --- |
| EGDe | Wild type (wt) strain, serovar 1/2a, Clonal complex 9 | BUG1600  (ATCC BAA-679) | - | ^1^ |
| EGDe Δ*inlP* | Deletion mutant for *inlP* | HBSC145 | - | This study |
| EGDe pP1 | EGDe carrying the empty pP1 plasmid | HBSC123 | Ery5 | ^2^ |
| EGDe pP1-*inlP*  (EGDe-InlP+) | EGDe expressing the *inlP* gene under the control of the pProt promoter | HBSC026 | Ery5 | This study |
| 10403S | Wild type (wt), serovar 1/2a, Clonal complex 7 | BUG1361 | - | ^1^ |
| 10403S pP1 | 10403S carrying the empty pP1 plasmid | HBSC124 | Ery5 | This study |
| 10403S pP1-*inlP*  (10403S-InlP+) | 10403S expressing the *inlP* gene under the control of the pProt promoter | HBSC017 | Ery5 | This study |
| 10403S Δ*inlP* | Deletion mutant for *inlP* | HBSC142 | - | This study |
| 10403S Δ*inlP^C-^* | Deletion mutant for *inlP* carrying the integrative empty pPL2 plasmid | HBSC332 | Cm7 | This study |
| 10403S Δ*inlP^inlP-^*^V5^  (*inlP*-_V5_ strain) | Deletion mutant for *inlP* carrying the integrative pAD-*^inlP-^*^V5^ | HBSC202 | Cm7 | This study |

Cm: chloramphenicol; Ery: erythromycin

The *inlP* deletion mutant strains (Δ*inlP*) were generated by allelic exchange, as described previously ^2^. The deletion of the *inlP* gene in EGDe-Δ*inlP* and 10403S-Δ*inlP* was confirmed by PCR amplification and sequencing.

The InlP-expressing strains (EGDe-InlP+) and (10403S-InlP+), and control strains carrying the empty vector pP1, were generated by electroporatation of plasmid pP1-*inlP,* or *pP1,* into EGDe or 10403S strains, respectively.

The *inlP-V5* strain carrying a chromosomal copy of *inlP*-V5 gene was generated by conjugation and integration of pAD-*inlP*-V5 plasmid into the genome of *L. monocytogenes* 10403S Δ*inlP*, generating strain 10403SΔ*inlP-inlP*-V5. An isogenic control strain was produced by integration of the empty pPL2 plasmid (10403S Δ*inlP*^C-^). The selected integrants were confirmed by PCR.

**1.2. Plasmids**

The bacterial plasmids used in this work are listed below.

| Plasmid | Hosts | Selection  µm/mL) | Characteristics | Reference |
| --- | --- | --- | --- | --- |
| pB27-*inlP* | *E. coli /Yeast* | Tet7 | pB27 carrying *inlP* (*lmo2470*)  (without the signal peptide) | This study |
| pB27- *lmo2027* | *E. coli /Yeast* | Tet7 | pB27 carrying *lmo2027*  (without the signal peptide) | This study |
| pP1 | *E. coli/L. m.* | Ery150 | pAT18 carrying the pProt promoter from the protease gene of *Streptococcus cremoris* | ^3^ |
| pP1-*inlP* | *E. coli/L. m.* | Ery150 | pP1 carrying *inlP,* under the control of the pProt promoter | This study |
| pPL2 | *E. coli/L. m.* | Cm30 | Integrative plasmid of *L.m.,* from which the pAD plasmid is derived | ^4^ |
| pAD-*inlP*-_V5_ | *E. coli/L. m.* | Cm30 | Integrative plasmid of *L. m.* carrying the gene *inlP-V5,* under the control of the P_HYPER_ promoter | This study |
| pGEX4T1-*inlP* | *E. coli* | Amp100 | pGEX4T1 carrying the gene *inlP* (codon optimized for *E. coli*) | This study  (GeneCust) |
| pGEX4T1-*lntA* | *E. coli* | Amp100 | pGEX4T1 carrying the gene *lntA* (codon optimized for *E. coli*) | ^5^ |
| pcDNA4-inlP-_HA-FLAG_ | *E. coli/*  Human cells | Amp100 | pcDNA4 carrying the gene *inlP* (codon optimized for *H. sapiens*) HA- and FLAG-tagged | This study  (GeneCust) |
| pcDNA4-lmo2027-_HA-FLAG_ | *E. coli/*  Human cells | Amp100 | pcDNA4 carrying the gene *lmo2027* (codon optimized for *H. sapiens*) HA- and FLAG-tagged | This study  (GeneCust) |
| pcDNA5- GFP-InlP | *E. coli/*  Human cells | Amp100 | pcDNA5-FRT-FA-PC-GFP-Blue carrying the gene *inlP* fused with the GFP | This study |
| pCMV6-RBM5-_Myc-FLAG_ | *E. coli/*  Human cells | Kan30 | pCMV6 carrying the gene *RBM5* Myc and FLAG-tagged | Origen |
| pCMV6  RBM5-ΔRRM1-2-_Myc-FLAG_ | *E. coli/*  Human cells | Kan30 | pCMV6 carrying the gene *RBM5* deleted for the RRM1, the Zinc finger domain and RRM2  Myc- and FLAG-tagged | This study  (GeneCust) |
|  |  |  |  |  |
| pCMV6  RBM5-ΔOCRE-_Myc-FLAG_ | *E. coli/*  Human cells | Kan30 | pCMV6 carrying the gene *RBM5* deleted for the OCRE domain  Myc- and FLAG-tagged | This study  (GeneCust) |

*L. m.*: *Listeria moncoytogenes*.

Amp, ampicillin; Cm: Chloramphenicol; Ery: Erythromycin; Kan: kanamycin; Tet: tetracycline

The plasmid pB27-*inlP* and pB27-*lmo2027* used for the Y2H screen were generated by cloning the sequence (in the *L. monocytogenes* EGDe strain genome) encoding InlP (aa 31-358) or Lmo2027 (aa 31-367) proteins without their signal peptide, in plasmid pB27 (Hybrigenics, S.A., Paris, France; http://www.hybrigenics.com) at the *Pac*I and *Spe*I restriction sites.

The constitutive expression plasmid pP1-*inlP* was constructed by cloning the *inlP* sequence (from ribosome binding site to stop codon) of *L. moncytogenes* EGDe DNA, after PCR-amplification using the following primers:

5’-ACGAGCTCAAAAGAAAGGACTGAATACATTGAG-3’

5’-ACATGCATGCTTATTAATTAATAGTTACATTCCAATC-3’.

The DNA fragment was inserted at the *Sac*I and *Sma*I sites of plasmid pP1 ^3^ downstream of the pProt promoter, generating pP1-*inlP*, which was electroporated into EGDe or 10403S strains.

The plasmid pPL2 P_HYPER_-5’-UTR_hly_-*inlP*-*V5* (or pAD-*inlP*-V5) was constructed into plasmid pAD-cGFP ^6^ after removal of the 5'-UTR*_hly_-gfp* insert. The 5’-UTR_hly_-*inlP*-*V5* sequence (entire10403S *inlP* reading frame fused with the 5'UTR of *hly* in N-terminal and a V5 tag in C-terminal) was synthesized by GeneCust (France) and then cloned at the *Eag*I and *Sal*I restriction sites, giving plasmid pAD-*inlP*-V5.

The expression vector pGEX4T1-*inlP* was generated by GeneCust (France) after synthesis of the *inlP* gene (without the signal peptide sequence) in fusion with an HA tag followed by two stop codons, with optimization of codon usage for *E. coli*, then insertion of the fragment at the *BamH*I and *Xho*I sites of plasmid pGEX-4T-1 (GE Healthcare).

The InlP-_HA-FLAG_ and Lmo2027-_HA-FLAG_ expression vectors were generated after synthesis of the *inlP* or *lmo2027* genes (without the signal peptide sequence) in fusion with HA and FLAG tags followed by two stop codons, with optimization of codon usage for human cells, then insertion of the fragments into pcDNA4/TO-Myc-his at the *BamH*I and *EcoR*I restriction sites.

The pcDNA5-GFP-*inlP* expression vector was generated by PCR-amplification of the cDNA coding for humanized *InlP* and N-terminally fused with the GFP sequence, followed by insertion in the pcDNA5-FRT-FA-PC-GFP-Blue vector ^7^ at the *Fse*I and *Asc*I restriction sites.

The RBM5-_Myc-FLAG_ expression vector is from OriGene Technologies (USA). The expression vectors RBM5ΔRRM1-2-_Myc-FLAG_ and ΔOCRE-_Myc-FLAG_ were generated by GeneCust (France).

**1.3. Western Blot**

Protein extracts were denatured at 95°C for 5 min in a final 1X Laemmli loading buffer, before being loaded into denaturing polyacrylamide gels of varying percentages depending on the experiments. After migration by electrophoresis, proteins were transferred in semi-dry medium to a nitrocellulose membrane using a transfer apparatus (Fast blotter PierceG2, Thermo) and the manufacturer's transfer buffer. The time and power parameters of this transfer were optimized according to the size of the proteins of interest and the percentage of polyacrylamide gel. When necessary, protein extracts were first analyzed by staining the membrane with Ponceau red, then rinsed with water and then with PBS-Tween 0.2% before the blocking step of 1h at room temperature in PBS-Tween 0.2% containing 5% non-fat milk powder. The membranes were incubated overnight with the primary antibody at 4°C. After three 10 min washes with PBS-Tween 0.2%, membranes were incubated for one hour in the presence of the secondary antibody coupled with horseradich peroxidase (HRP) enzyme (1:10000) and washed three more times with PBS-Tween 0.2% before the proteins of interest were revealed by chemiluminescence (Millipore kit, western immobilization) and observed with Chemidoc Biorad.

**1.4. Image quantification**

Quantification of the number of apoptotic cells was performed by observation of condensed or fragmented nuclei in Hoechst staining, according to the protocol described by Fuschimi et al. (2008) ^8^, for three biological replicates (n=30 cells per replicate, from three independent technical replicates). The quantification of nuclei containing RBM5 nuclear condensates was performed by analysis of nuclear bodies darker than nucleoli in phase contrast microscopy and surrounded by an RBM5-Myc ring, in 3 biological replicates (n=25 cells per replicas). SC35 speckles roundness were calculated with Image J software ^9^. Speckles from 20 nuclei per group were segmented using the Otsu thresholding method ^10^. Speckles circularity was then assessed with the formula: *circularity = 4pi(area/perimeter²).* A circularity value of 1.0 indicates a perfect circle. As the value approaches 0, it indicates an increasingly elongated polygon.

**1.5. Transcriptomic data of *L. monocytogenes* gene expression during infection of cultured cells or mice**

The mRNA transcriptional profile of intracellular *L. monocytogenes* 6 hours after infection of Caco-2-epithelial cells or 8 hours after infection of P388D1 murine macrophage, compared to growth in BHI medium was assessed by Joseph et al. (2006) and Schultze et al. (2015), respectively ^11^ ^12^. Camejo et al (2009) performed an *in vivo* study that highlighted the transcriptional profile of *L. monocytogenes* in murine spleens at 24, 48, and 72 hours post-intravenous infection, compared to growth in BHI medium ^13^.

**1.6. Supplementary References**

1 Becavin, C. *et al.* Comparison of widely used Listeria monocytogenes strains EGD, 10403S, and EGD-e highlights genomic variations underlying differences in pathogenicity. *MBio* **5**, e00969-00914, doi:10.1128/mBio.00969-14 (2014).

2 Sabet, C. *et al.* The Listeria monocytogenes virulence factor InlJ is specifically expressed in vivo and behaves as an adhesin. *Infect Immun* **76**, 1368-1378, doi:10.1128/IAI.01519-07 (2008).

3 Dramsi, S. *et al.* Entry of Listeria monocytogenes into hepatocytes requires expression of inIB, a surface protein of the internalin multigene family. *Molecular microbiology* **16**, 251-261 (1995).

4 Lauer, P., Chow, M. Y., Loessner, M. J., Portnoy, D. A. & Calendar, R. Construction, characterization, and use of two Listeria monocytogenes site-specific phage integration vectors. *J Bacteriol* **184**, 4177-4186, doi:10.1128/JB.184.15.4177-4186.2002 (2002).

5 Lebreton, A. *et al.* Structural basis for the inhibition of the chromatin repressor BAHD1 by the bacterial nucleomodulin LntA. *MBio* **5**, e00775-00713, doi:10.1128/mBio.00775-13 (2014).

6 Balestrino, D. *et al.* Single-cell techniques using chromosomally tagged fluorescent bacteria to study Listeria monocytogenes infection processes. *Appl Environ Microbiol* **76**, 3625-3636, doi:10.1128/AEM.02612-09 (2010).

7 Helfer, E. *et al.* Endosomal recruitment of the WASH complex: active sequences and mutations impairing interaction with the retromer. *Biol Cell* **105**, 191-207, doi:10.1111/boc.201200038 (2013).

8 Fushimi, K. *et al.* Up-regulation of the proapoptotic caspase 2 splicing isoform by a candidate tumor suppressor, RBM5. *Proc Natl Acad Sci U S A* **105**, 15708-15713, doi:10.1073/pnas.0805569105 (2008).

9 Schneider, C. A., Rasband, W. S. & Eliceiri, K. W. NIH Image to ImageJ: 25 years of image analysis. *Nat Methods* **9**, 671-675, doi:10.1038/nmeth.2089 (2012).

10 Otsu, N. A threshold selection method from gray-level histograms. *EEE Trans Sys Man Cyber.* **9**, 62-66 (1979).

11 Joseph, B. *et al.* Identification of Listeria monocytogenes genes contributing to intracellular replication by expression profiling and mutant screening. *J Bacteriol* **188**, 556-568, doi:10.1128/JB.188.2.556-568.2006 (2006).

12 Schultze, T. *et al.* A detailed view of the intracellular transcriptome of Listeria monocytogenes in murine macrophages using RNA-seq. *Front Microbiol* **6**, 1199, doi:10.3389/fmicb.2015.01199 (2015).

13 Camejo, A. *et al.* The arsenal of virulence factors deployed by Listeria monocytogenes to promote its cell infection cycle. *Virulence* **2**, 379-394, doi:10.4161/viru.2.5.17703 (2011).

**Supplementary Figures**


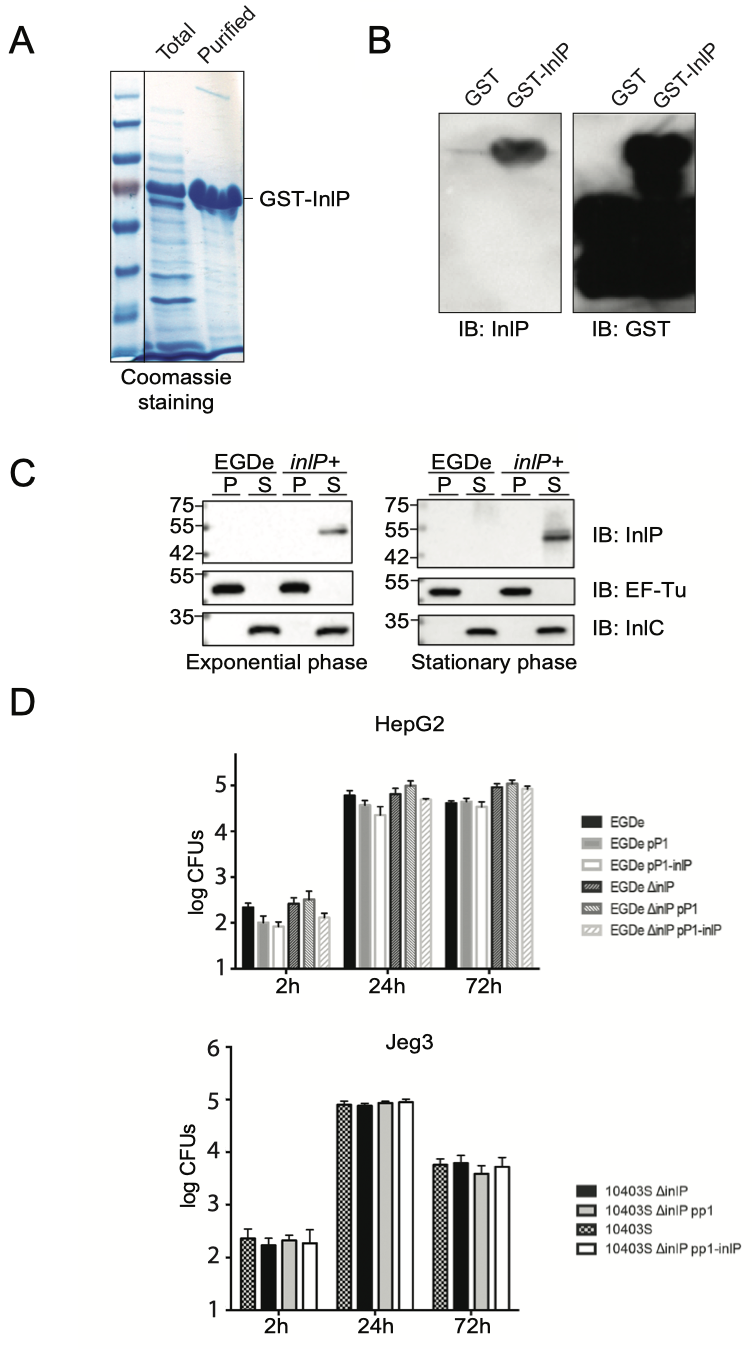


**Figure S1. InlP is not expressed *in vitro* and its absence does not alter bacterial loads upon intracellular infection of *L. monocytogenes* in human HepG2 or JEG3 cells.**

**(A)** Coomassie staining of crude *E. coli* extract induced with IPTG (see methods) and affinity purified GST-InlP. **(B)** Western blotting analysis with an anti-InlP purified antibody (see method, left panel) or an anti-GST antibody (right panel) on purified GST and GST-InlP. (**C**) Analysis of InlP production in bacterial culture pellets and supernatants from *L. monocytogenes* EGDe containing plasmid pP1 (EGDe) or pP1-*inlP (inlP+)* grown at exponential phase (OD_600_ of 0.5) or until the stationary phase*.* (**D**) Infection assays in human hepatocytes HepG2 or placental JEG-3 cells. The number of intracellular bacteria of each tested strain of *L. monocytogenes* (indicated according to the legend in the boxes on the right) was assessed by spreading serial dilutions of cell lysates on BHI agar plates at the indicated time point. Histograms represent the mean ± standard deviation of Log colonies forming units (CFUs) per ml, from three experiments.


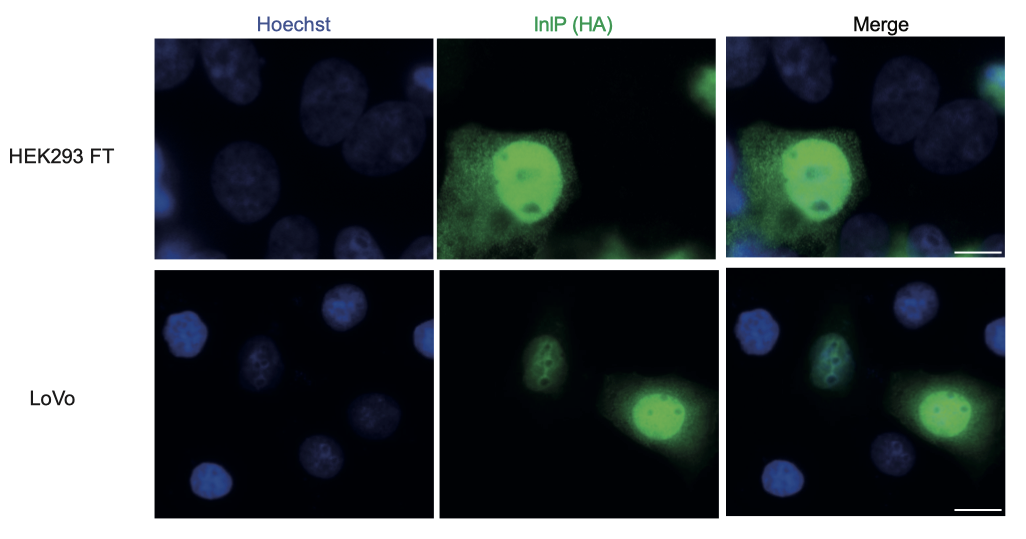


**Figure S2. InlP localizes to the nucleus in HEK293 FT and LoVo cells.**

Representative immunofluorescence images of InlP-_HA_ localization in HEK293 FT and LoVo transfected cells. InlP-_HA_ is detected with a HA antibody and the nucleus with Hoechst.

**
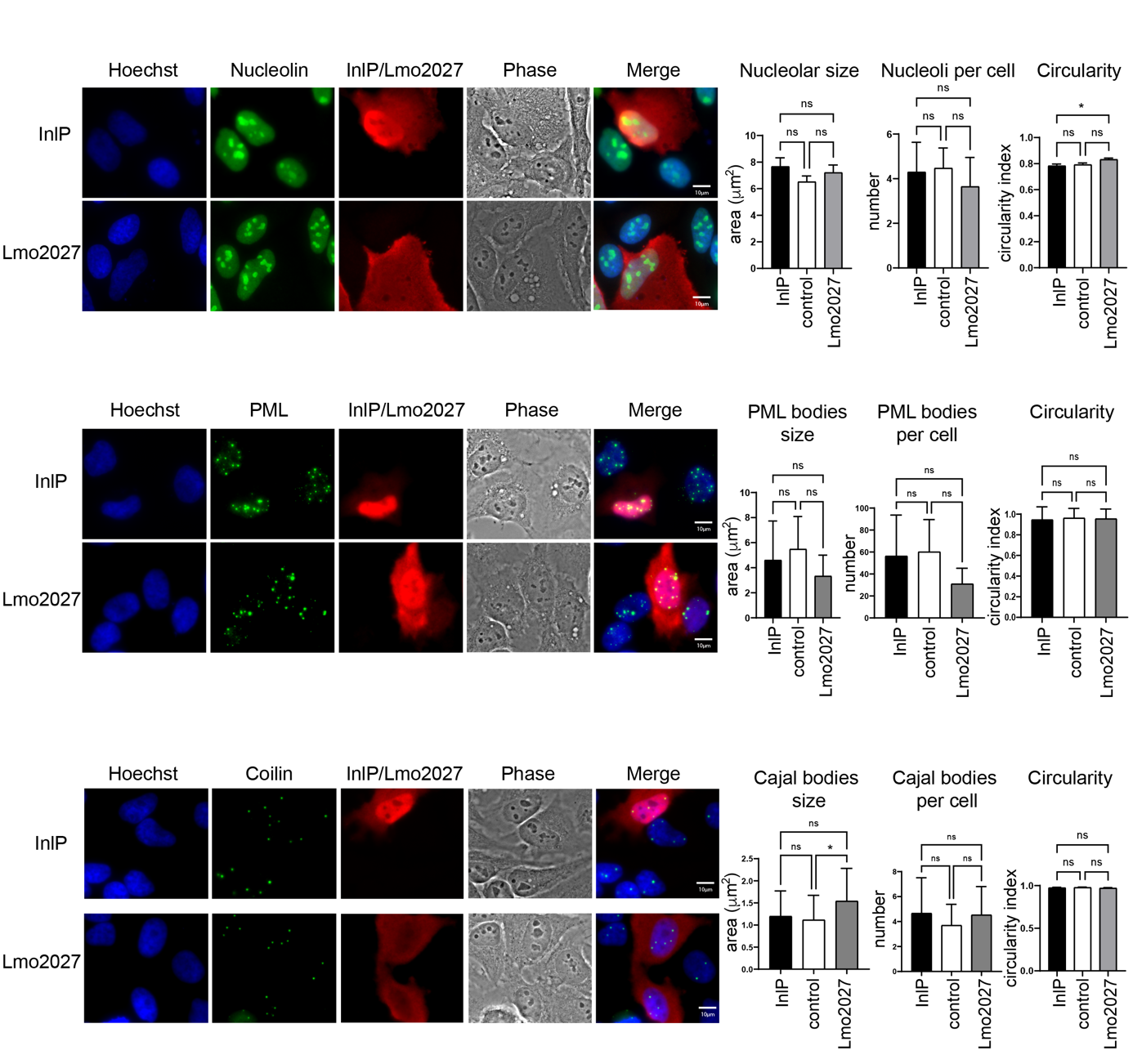
**

**Figure S3. InlP does not affect the structures of a set of nuclear bodies.**

Left, representative immunofluorescence images of HeLa cells transfected with InlP-_HA_, Lmo2027-_HA_ and stained with various markers of nuclear bodies (nucleolin for the nucleoli; PML for PML bodies, Coilin for Cajal bodies), as indicated. Scale bars, 10 µm. Right, quantification of the size, number and circularity of each nuclear body in untransfected cells (control) or cells transfected with either InlP or Lmo2027, as indicated.


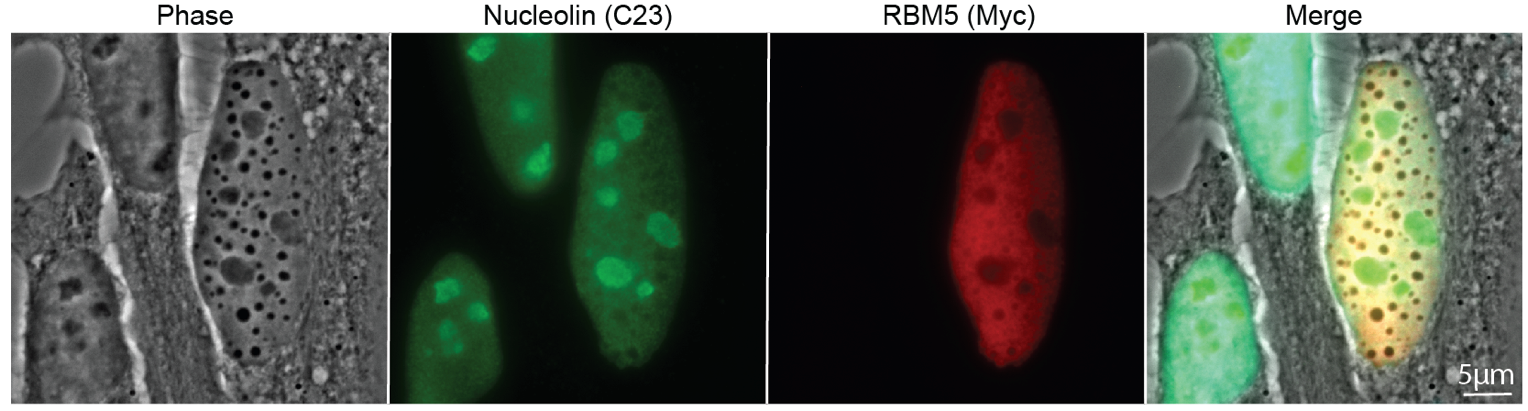


**Figure S4. RBM5 granules do not colocalize with nucleoli.**

Representative immunofluorescence image of HeLa cells cotransfected with RBM5-_Myc_ and InlP-_HA_ and immunolabeled with Myc and nucleolin antibodies. Scale bars: 5μm.


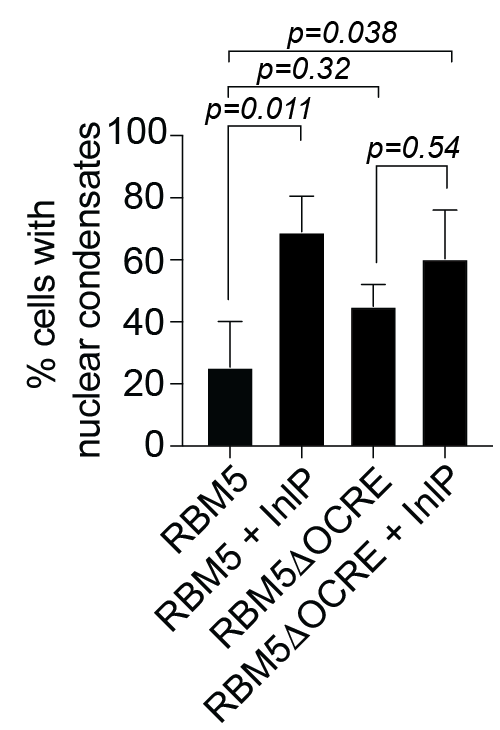


**Figure S5. The InlP-mediated stimulation of RBM5 granules biogenesis requires the OCRE domain of RBM5.**

Nuclei with RBM5-condensates were scored in RBM5- or RBM5ΔOCRE-expressing HeLa cells, in absence or presence of InlP. Histograms represent percentage of each category as mean ± SD of 3 independent experiments. Statistical significance determined by ANOVA.
